# Electron-Driven Self-Assembly of Salt Nanocrystals in Liquid Helium**

**DOI:** 10.1002/anie.201409465

**Published:** 2014-11-06

**Authors:** Matthias Daxner, Stephan Denifl, Paul Scheier, Andrew M Ellis

**Affiliations:** Institut für Ionenphysik und Angewandte Physik, Universität InnsbruckTechnikerstrasse 25, 6020 Innsbruck (Austria); Department of Chemistry, University of Leicester, University Road, LeicesterLE1 7RH (UK)

**Keywords:** electron transfer, helium nanodroplets, mass spectrometry, salt nanocrystals, sodium

## Abstract

The self-assembly of salt nanocrystals from chemical reactions inside liquid helium is reported for the first time. Reaction is initiated by an electron impacting a helium nanodroplet containing sodium atoms and SF_6_ molecules, leading to preferential production of energetically favorable structures based on the unit cell of crystalline NaF. These favorable structures are observed as magic number ions (anomalously intense peaks) in mass spectra and are seen in both cationic and anionic channels in mass spectra, for example, (NaF)_*n*_Na^+^ and (NaF)_*n*_F^−^. In the case of anions the self-assembly is not directly initiated by electrons: the dominant process involves resonant electron-induced production of metastable electronically excited He^−^ anions, which then initiate anionic chemistry by electron transfer.

Neutral and ionic alkali-metal halide clusters have been widely studied, both experimentally and theoretically. Part of the motivation to study these species is to see if the three-dimensional structures of the crystalline salts are retained in relatively small clusters. Such information can be derived from mass spectrometry through the observation of anomalously intense peaks (so-called magic number features). Several methods have been used to produce alkali-metal halide cluster ions in the gas phase, including sputtering,[1] laser ablation,[2] electrospray,[3] and ion–molecule reactions in a flowing afterglow.[4] The general finding, whether detecting cations or anions, is that enhanced signal intensity is seen for cluster ions (magic-number ions) of composition consistent with one or more complete unit cells. On these grounds it is reasonable to suppose that the cluster ions adopt structures based on the normal crystalline structure of the extended solid.

Herein we show that it is possible to form alkali-metal halide clusters by reactions between clusters of sodium and SF_6_ in liquid helium nanodroplets. The low intrinsic temperature (ca. 0.4 K) and the rapid cooling of dopants in these droplets,[5] which is assisted by the high thermal conductivity of superfluid helium, should inhibit chemical reactions. However, reaction between sodium and SF_6_ can be triggered by electron impact on the droplet, leading to a rich range of cationic and anionic salt clusters. Particularly surprising is that self-assembly into structures based on the unit cell of NaF occurs even when the chemistry is initiated inside a liquid helium nanodroplet.

The two reagents were added separately to the helium droplets, with sodium vapor coming from an oven containing solid sodium while the SF_6_ was supplied from a gas cylinder. SF_6_ was added to the first pick-up cell and sodium vapor to the second pick-up cell. The partial pressures of the dopants were set so that the most probable process was pick-up of a relatively small number of dopant atoms/molecules. However, the statistical nature of the pick-up process means that, in practice, clusters spanning a relatively wide range of sizes were generated. Reaction products derived from electron injection into the droplets were detected in the gas phase using mass spectrometry.

The major cationic products can be divided into two types: (NaF)_*n*_Na^+^ and (NaF)_*n*_(Na_2_S)_*m*_Na^+^. By way of contrast the stoichiometric (NaF)_*n*_^+^ cluster ions are more than an order of magnitude less abundant than the (NaF)_*n*_Na^+^ ions. A small quantity of (NaF)_*n*_Na^+^ ions with added helium atoms were also observed, as can be seen in Figure 1. The survival of these adduct ions suggests that at least some of the cluster ions produced are ultimately cold enough to retain one or more helium atoms.

**Figure 1:**
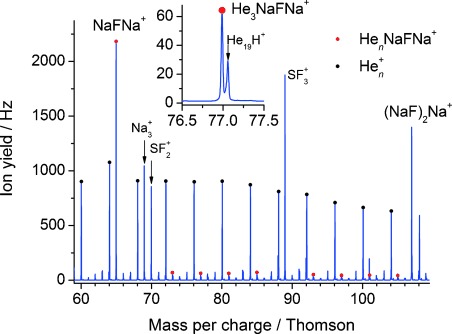
A section of the cation mass spectrum obtained from electron-induced reactions between Na_*n*_ and (SF_6_)_*m*_ clusters. This mass spectrum was recorded at an electron energy of 100 eV.

The upper trace of Figure 2 summarizes the measured abundance of (NaF)_*n*_Na^+^ as a function of 2 *n*+1, the number of atoms in the cluster ion. A relatively smooth decline is seen in the ion signal as *n* increases, which reflects the fact that the experimental conditions were chosen to bias the maximum pick-up probability to a relatively small number of dopant atoms/molecules, as mentioned earlier. This smooth decline is punctuated by clear magic number peaks corresponding to *n*=4, 13, 22, and 37. These magic number ions are well-known from earlier studies in the gas phase[6] and those at *n*=13, 22 and 37 correspond to structures consisting of one, two, and four complete units cells of sodium fluoride, which are especially stable because they maximize the attractive Coulombic interactions between the constituent ions. The ions at *n*=31, which correspond to a cluster composed of three complete unit cells, show a marginal increase in abundance against the downward trend but do not show obvious magic character. Calculations have shown that the most stable structure of the *n*=4 cluster is a non-planar 3×3×1 sheet with an Na^+^ ion at the center.[7–9]

**Figure 2:**
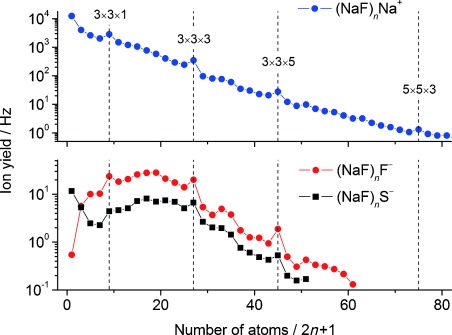
Abundance plots for the major cationic (upper trace) and anionic (lower traces) products. The labels *x*×*y*×*z* refer to the number of ions located along Cartesian coordinates, for example, 3×3×3 refers to the cubic unit cell of sodium fluoride.

Also seen are cations which incorporate the sulfur from SF_6_, namely (NaF)_*n*_(Na_2_S)_*m*_Na^+^, with *m*≤4. One possibility is that these ions contain SF_*x*_ remnants from incomplete reaction of SF_6_ with Na_*n*_. However, the very specific stoichiometries observed suggest complete reaction, leading to a mixed salt containing both Na_2_S and NaF units, which is unknown in the solid state. Magic numbers can be seen for these ions but there is no obvious pattern and we are unable to extract meaningful structural information from these enhanced peaks at the present time.

In addition to cations, anionic salt clusters were also observed. (NaF)_*n*_^−^ ions were minor products, being some two orders of magnitude less abundant than the main anions seen, (NaF)_*n*_F^−^ and (NaF)_*n*_S^−^. The lower trace in Figure 2 summarizes the relative abundances of (NaF)_*n*_F^−^ and (NaF)_*n*_S^−^ as a function of 2 *n*+1. Although there are differences in the overall shapes of the ion distribution curves, the magic numbers match those seen for the (NaF)_*n*_Na^+^ cations. For (NaF)_*n*_F^−^ this is expected from earlier work[4,10] and is consistent with geometric rather than electronic structure being the key for determining the most stable ions. For (NaF)_*n*_S^−^, which has not been detected previously, it would seem reasonable to assume that sulfur is present as S^−^ rather than S^2−^ and merely substitutes directly for the F^−^, leading to the same structural behavior. In fact DFT calculations on (NaF)_*n*_S^−^ clusters confirm this assumption and full details will be presented in a subsequent publication.

Figure 3 shows the dependence of the signal on the electron energy for two illustrative cations and anions, (NaF)_4_Na^+^ and (NaF)_4_F^−^, along with the signal recorded for the He_9_^+^ cluster ion. Similar curves are obtained for other comparable ions and these provide information on how the charged salt clusters are formed in the helium droplets. Cations can be made either by Penning ionization,[11] which involves collision of a dopant with a metastable electronically excited helium atom (threshold 19.8 eV in the gas phase owing to production of the 2 ^3^S_1_ metastable state of helium, which we write in shorthand notation as He*) or by charge transfer from He^+^,[12–15] where the He^+^ has an energy onset of approximately 24.6 eV corresponding to the first ionization energy of a helium atom. The shape of the ion yield curve for (NaF)_4_Na^+^ in Figure 3 shows that both mechanisms occur: the low energy rise is derived solely from Penning ionization and after a short plateau charge transfer from He^+^ begins and the ion yield curve starts to resemble that seen for helium cluster cations, such as He_9_^+^.

**Figure 3:**
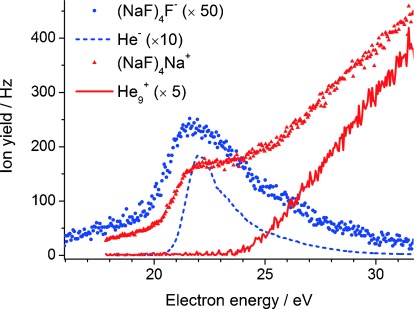
Signal level as a function of electron energy for (NaF)_4_F^−^, (NaF)_4_Na^+^, He_9_^+^, and He^−^. Note that the signals for (NaF)_4_F^−^, He_9_^+^, and He^−^ are expanded vertically relative to that of (NaF)_4_Na^+^.

The anion curve shows a resonance with an onset near 20 eV and which peaks at approximately 22 eV. This is consistent with a mechanism for anion formation initiated by He*. Note that the peak is broad and asymmetric on the high energy side, presumably owing to contributions from other metastable electronically excited states of helium above the 2 ^3^S_1_ state. However, there is no obvious way that neutral He* can generate anionic products. Instead, the production of anions represented in Figure 3 is presumed to derive from electron transfer from He^−^, whose production requires nearly the same energy as the formation of He*. He^−^ has only recently been identified as a product from electron impact of helium droplets.[16] Although atomic helium has a negative electron affinity in its ground state and therefore cannot bind an electron, excitation to the 2 ^3^S_1_ metastable state creates a far more polarizable entity with a small positive electron affinity, with the outermost electron bound to He* by 77 meV.[17] Recent work has shown that He^−^ is a highly mobile electron donor within a helium droplet.[16,18] The ion yield curve for (NaF)_4_F^−^ matches that of He^−^ (which is also shown in Figure 3) very closely and we conclude that He^−^ is the dominant source of anions in the current study. The anion peaks occur at approximately 22 rather than 20 eV because additional energy is required for an electron to enter a helium droplet.[19]

Also observed, but not shown in Figure 3, is a second anion resonance with a maximum near 2 eV and which is more than an order of magnitude weaker than the 22 eV feature. The 2 eV resonance is assigned to dissociative electron attachment to SF_6_, producing SF_5_^−^+F. This process occurs at 0.2 eV in the gas phase[20,21] but, like the He* production, is shifted to higher energy in liquid helium. The SF_5_^−^ ion must undergo reaction with Na_*n*_ and the subsequent chemistry is driven by the exothermicity of salt formation, leading to species such as (NaF)_4_F^−^.

In contrast to a study of Cs_*n*_ clusters with (H_2_O)_*m*_ clusters in helium droplets, where evidence was presented for an essentially barrierless reaction of the neutral reagents,[22] we believe that no reaction occurs between Na_*n*_ and (SF_6_)_*m*_ in helium nanodroplets prior to electron addition. Our reasoning is as follows. First, an activation barrier of 250 meV is known for the Na+SF_6_→NaF+SF_5_ reaction in the gas phase[23] which will be insurmountable at 0.4 K without an additional source of energy. Second, any reaction between Na_*n*_ and (SF_6_)_*m*_ will be highly exothermic and requires the dissipation of many eV of excess energy. Under these conditions neutral reaction products are likely to be ejected into the gas phase, which would then show ion-yield curves characteristic of the isolated neutral products. However, there is no evidence for ejected neutral products from the ion-yield data. The high reaction exothermicity might even generate ionic reaction products, but none were detected when the electron filament was turned off. Finally, the salt anion resonances match those of SF_6_ (very weak) and He*, whereas very different anion yield curves would be expected for direct electron attachment to neutral salt clusters. This combination of reasons provides strong evidence in favor of electron-initiated chemistry.

SF_6_ is known to reside inside helium droplets.[12,24] On the other hand alkali-metal atoms and small alkali-metal clusters sit in a dimple on the surface of a helium droplet because the repulsive interaction between the dopant and the helium is too large to allow an interior location.[25] We have shown elsewhere that once Na_*n*_ clusters reach a threshold size (*n*≥22) the clusters can now move inside a helium droplet.[26] When both (SF_6_)_*m*_ and Na_*n*_ clusters are combined in a helium droplet the substantial polarizability of SF_6_ will create an attractive (dispersive) interaction with Na_*n*_. We therefore anticipate that clusters smaller than Na_22_ will now relocate to the droplet interior when (SF_6_)_*m*_ is also present and, although we have no specific evidence, this may even occur for atomic sodium. Thus, instead of segregated reagents which come into contact after electron impact, the likelihood is that the two reagents are directly in contact and frozen in place prior to the electron entering the droplet.

In summary, this study has shown that salt clusters can be formed by chemistry initiated by electron impact on helium droplets containing Na_*n*_ and (SF_6_)_*m*_. Mass spectra reveal that the product cations and anions self-assemble into the classic face-centered cubic structure of alkali-metal halide solids, with clusters based on complete unit cells showing added stability. An important observation is that the dominant source of anions is reaction with metastable He^−^. We anticipate that this mobile charge carrier will be a rich source of anion chemistry in helium nanodroplets.

## Experimental Section

Helium nanodroplets were produced by expanding high-purity helium gas at a stagnation pressure of 20 bar and a temperature of 9.4 K through a 5 μm pinhole into a vacuum. Under these conditions the average number of helium atoms per droplet was ca. 10^5^. After being skimmed to form a collimated beam the droplets passed through two consecutive pick-up cells, the first of which was used to add SF_6_ (Ausimont, 99.9 % purity) and the second to add sodium atoms (Sigma Aldrich, 99.9 %). The sodium was generated by evaporation of the corresponding solid at a temperature of 120 °C. After dopant pick-up the droplets entered another differentially pumped chamber and were exposed to an electron beam of variable energy (0—150 eV). Any ions produced were then extracted into a commercial (Tofwerk) time-of-flight (ToF) mass spectrometer with a mass resolution of ca. 5000 for positive ions and 2000 for negative ions.

## References

[1] Campana JE, Barlak TM, Colton RJ, DeCorpo JJ, Wyatt JR, Dunlap BI (1981). Phys. Rev. Lett.

[2] Fatemi FK, Dally AJ, Bloomfield LA (2003). Phys. Rev. Lett.

[3] Wang G, Cole RB (1998). Anal. Chem.

[4] Miller TM, Lineberger WC (1990). Int. J. Mass Spectrom. Ion Process.

[5] Toennies JP, Vilesov AF (2004). Angew. Chem. Int. Ed.

[6] Martin TP (1983). Phys. Rep.

[7] Diefenbach J, Martin TP (1985). Surf. Sci.

[8] Lintuluoto M (2001). J. Mol. Struct. THEOCHEM.

[9] Aguado A, Ayuela A, Lopez JM, Alonso JA (1998). Phys. Rev. B.

[10] Campana JE, Dunlap BI (1984). Int. J. Mass Spectrom. Ion Process.

[11] Schöbel H, Bartl P, Leidlmair C, Daxner M, Zöttl S, Denifl S, Märk TD, Scheier P, Spångberg D, Mauracher A, Bohme DK (2010). Phys. Rev. Lett.

[12] Scheidemann A, Schilling B, Toennies JP (1993). J. Phys. Chem.

[13] Callicoatt BE, Förde K, Ruchti T, Jung L, Janda KC (1998). J. Chem. Phys.

[14] Lewis WK, Lindsay M, Bemish RJ, Miller RE (2005). J. Am. Chem. Soc.

[15] Ellis AM, Yang S (2007). Phys. Rev. A.

[16] Mauracher A, Daxner M, Postler J, Huber SE, Denifl S, Scheier P, Toennies JP (2014). J. Phys. Chem. Lett.

[17] Kristensen P, Pedersen UV, Petrunin VV, Andersen T, Chung KT (1997). Phys. Rev. A.

[18] A. Mauracher, M. Daxner, S. E. Huber, J. Postler, M. Renzler, S. Denifl, P. Scheier, A. M. Ellis, *Angew. Chem. Int. Ed**Angew. Chem*

[19] Denifl S, Zappa F, Mähr I, Lecointre J, Probst M, Märk TD, Scheier P (2006). Phys. Rev. Lett.

[20] Fenzlaff M, Gerhard R, Illenberger E (1988). J. Chem. Phys.

[21] Braun M, Ruf MW, Hotop H, Allan M (2006). Chem. Phys. Lett.

[22] Müller S, Krapf S, Koslowski Th, Mudrich M, Stienkemeier F (2009). Phys. Rev. Lett.

[23] Düren R, Färber M, Heumann B, Knepper M, Mohr S, Weiss C, Te Lintel Hekkert S, Linskins AF, Reuss J (1996). J. Chem. Phys.

[24] Hartmann M, Miller RE, Toennies JP, Vilesov A (1995). Phys. Rev. Lett.

[25] Stienkemeier F, Higgens J, Callegari C, Kanorsky SI, Ernst WE, Scoles G (1996). Z. Phys. D.

[26] An der Lan L, Bartl P, Leidlmair C, Schöbel H, Jochum R, Denifl S, Märk TD, Ellis AM, Scheier P (2011). J. Chem. Phys.

